# How genetics can help diagnosis and treatment in psychiatric conditions

**DOI:** 10.1192/j.eurpsy.2021.156

**Published:** 2021-08-13

**Authors:** B. Chaumette, C. Laurent-Levinson, P. Almos, F. Degenhardt

**Affiliations:** 1 Inserm U1266, Institute of Psychiatry and Neuroscience of Paris, Paris, France; 2 Crmr Psychiatrie, GHU Paris Psychiatrie et Neurosciences, Paris, France; 3 Groupe De Recherche Clinique N°15 - Troubles Psychiatriques Et Développement (psydev), Faculté de Médecine Sorbonne Université, Paris, France; 4 Centre De Référence Des Maladies Rares à Expression Psychiatrique, Department Of Child And Adolescent Psychiatry, AP-HP, Hôpital Universitaire de la Pitié-Salpêtrière, Paris, France; 5 Department Of Psychiatry, Faculty Of Medicine, University of Szeged, Szeged, Hungary; 6 Department Of Child And Adolescent Psychiatry, Psychosomatics and Psychotherapy Institute, Essen, Germany

**Keywords:** molecular diagnosis, rare diseases, genetics

## Abstract

The understanding of the genetic architecture of psychiatric disorders has made significant advances in the last decade and some scientific findings can now be translated into clinical practice. The rise of genetic testing and the awareness of patients and their families motivate psychiatrists to examine this approach. The COST Action EnGagE (CA17130) is promoting these developments in Europe. Whereas the findings of common variants are the domain of research, screening for rare variants at the genome-wide level is already applicable in clinical practice. It is now possible to return meaningful results to the individual to help him/her understanding the disease and the comorbidities, to guide treatment and to perform genetic counseling. In this presentation, we will give meaningful examples for psychiatric practice. For instance, around one-third of the patients diagnosed with autism spectrum disorder can benefit from a molecular diagnosis (fragile X syndrome, SHANK3 deletion…). Microdeletion or microduplication may explain a fraction of schizophrenic cases (e.g. del22q11). Identification of rare variants causing the disease may decrease the stigma and feeling of guilt often reported by patients and families. This could also help to detect and manage other comorbidities. It is expected that treatment guidelines and clinical trials would be developed in the near future for patients carrying a rare variant, opening the way to personalized psychiatry. Finally, this effort has a huge impact on the family, by enhancing genetic counseling in psychiatry. The rise of psychiatric genetics might align our field more closely with the other medical specialties.

**Disclosure:**

No significant relationships.

